# Long Non−Coding RNA H19 Regulates Glioma Cell Growth and Metastasis *via* miR-200a-Mediated CDK6 and ZEB1 Expression

**DOI:** 10.3389/fonc.2021.757650

**Published:** 2021-11-02

**Authors:** Xuezhu Chen, Yuhong Li, Chenghai Zuo, Kaiyuan Zhang, Xuejiao Lei, Ju Wang, Yang Yang, Jianmin Zhang, Kang Ma, Shi Wang, Ning Mu, Chuanyan Yang, Jishu Xian, Hua Feng, Rongrui Tang, Tunan Chen

**Affiliations:** ^1^ Department of Neurosurgery and Key Laboratory of Neurotrauma, Southwest Hospital, Third Military Medical University (Army Medical University), Chongqing, China; ^2^ Department of Neurosurgery, General Hospital of Xinjiang Military Command of People’s Liberation Army (PLA), Urumqi, China; ^3^ Department of Neurosurgery, The 904^th^ Hospital of People’s Liberation Army (PLA), School of Medicine of Anhui Medical University, Wuxi, China; ^4^ Department of Neurosurgery, University-Town-Hospital of Chongqing Medical University, Chongqing, China

**Keywords:** glioma, H19, miR-200a, metastasis, proliferation, invasion

## Abstract

Long non-coding RNAs (lncRNAs) serve essential roles on various biological functions. Previous studies have indicated that lncRNAs are involved in the occurrence, growth and infiltration of brain tumors. LncRNA H19 is key regulator in the pathogenesis of gliomas, but the underlying mechanisms of H19-regulated tumor progression remain unknown. Therefore, we investigated the effects and mechanism of action of lncRNA H19 on the homeostasis of glioma cells. As a novel oncogenic factor, up-regulation of H19 was able to promote the proliferation of glioma cells by targeting miR-200a. Furthermore, elevated miR-200a levels could reverse H19-induced cell growth and metastasis. Overexpression of miR-200a could significantly suppress the proliferation, migration and invasion of glioma cells. These biological behavior changes in glioma cells were dependent on the binding to potential target genes including CDK6 and ZEB1. CDK6 could promote cell proliferation and its expression was remarkably increased in glioma. In addition, up-regulation of miR-200a lead to reduction of CDK6 expression and inhibit the proliferation of glioma cells. ZEB1 could be a putative target gene of miR-200a in glioma cells. Thus, miR-200a might suppress cell invasion and migration through down-regulating ZEB1. Moreover, overexpression of miR-200a resulted in down-regulation of ZEB1 and further inhibited malignant phenotype of glioma cells. In summary, our findings suggested that the expression of H19 was elevated in glioma, which could promote the growth, invasion and migration of tumor cells *via* H19/miR-200a/CDK6/ZEB1 axis. This novel signaling pathway may be a promising candidate for the diagnosis and targeted treatment of glioma.

## Introduction

Glioma is a common type of malignancy in brain. It is characterized by poor prognosis and accounts for >90% of all intracranial tumors (1, 2). Recently, the diagnosis and treatment of glioma have been remarkably improved. However, the detailed molecular mechanisms involved in the progression of glioma remain unclear, so the prevention and treatment of this disease are still not satisfactory (3). Furthermore, it is well established that most tumors are highly polygenic, and proliferation and invasion of tumor cells are associated with the up-regulation of oncogenes or down-regulation of tumor suppressor genes (4, 5). Therefore, a better understanding of the molecular mechanisms involved in the pathogenesis of glioma is essential for the diagnosis, treatment and prognosis of this disease.

Until now, ~21,000 protein-coding genes have been identified in human genome, accounting for <2% of the entire genome sequence. Additionally, thousands of genes are transcribed into non-coding RNAs (ncRNAs) (6, 7). Recent studies have indicated that aberrantly expressed ncRNAs are associated with tumorigenesis, and numerous ncRNAs are key regulators of biological functions and disease pathogenesis (8, 9). LncRNAs are a group of non-coding RNAs whose length are >200 nucleotides. Recently, the regulatory functions of lncRNAs in tumor progression have been investigated in the field of RNA biology and transcriptomics. Among these lncRNAs, H19 was one of the first identified genes, and it is located on chromosome 11p15 (10, 11). Accumulating evidence have suggested that the levels of H19 are increased in a numerous type of malignancies, such as esophageal, colon, hepatocellular and bladder cancer (12–16). What’s more, H19 was also found to be significantly overexpressed in glioma cells, and its expression increased with the degree of malignancy (17, 18). One recent study demonstrated that H19 promoted glioma cells proliferation, migration, and angiogenesis *in vivo* (19). Therefore, these findings have indicated that H19 serves crucial roles in glioma progression. To data, the mechanisms are still largely unknown.

MicroRNAs (miRNAs) are a group ncRNAs with ~18-24 nucleotides. MiRNAs can negatively regulate the expression of target genes by interacting with correspondent mRNAs at the 3’-non-translation region (3’-UTR). Since numerous mRNAs are involved in cell migration and invasion, miRNA could potentially lead to the degradation or translation inhibition of these transcripts, subsequently participating in the regulation of tumor progression (20). Emerging evidence has indicated that miRNAs are key regulators of tumor progression through modulating cell growth, apoptosis, proliferation, migration and invasion. Furthermore, previous studies have revealed that miR-200a is a novel target of H19 in colon cancer. In addition, miRNA-200a could act as tumor suppressor as its levels are notably reduced in a variety of tumors, such as nasopharynx (21), liver (22) and ovarian cancer (23). However, the detailed roles of miR-200a in glioma are still unclear and require further investigation.

There are many functional downstream targets of miR-200a that mediate tumor progression. Among them, cyclin-dependent kinase 6 (CDK6) and ZEB1/ZEB2 were essential regulators in cell cycle and metastasis of tumor cells, respectively. CDK6 can promote cells to transit from G1 to S phase by activating the transcription of downstream genes involved in cell cycle regulation (24). Bioinformatic analyses have confirmed the potential binding sites of CDK6 on miR-200a, which functions as a negative regulator of CDK6. Up-regulation of CDK6 could abrogated the biological behavior changes in glioma cells caused by miR-200a. Additionally, previous studies have suggested that miR-200a could promote epithelial-mesenchymal transformation (EMT) and metastasis of tumor cells *via* targeting downstream genes ZEB1/ZEB2 (25–27). However, the biological effects of H19, miR-200a, CDK6 and ZEB1 as well as their interaction in glioma has not been elucidated.

In this study, the expression of H19, miR-200a, CDK6 and ZEB1 as well as their interaction in glioma have been investigated. Furthermore, the biological effects of H19, miR-200a, CDK6 and ZEB1 in glioma tissues/cells were also elucidated *in vivo* and *in vitro*. In summary, the regulatory roles of H19 in glioma have been revealed, which provides novel insights on the therapeutic development by inhibiting the migration and invasion of glioma cells.

## Materials and Methods

### Clinical Specimens and Tissue Microarray

A total of 15 paired glioma and non-tumour samples (≥5 cm from tumor margin) were obtained at Southwest Hospital (Chongqing, China), and written informed consent was obtained from all of the patients. The tissues were sectioned and snap-frozen in liquid nitrogen, then stored at -80°C until further use. The study was approved by the Ethics Committee of Southwest Hospital.

### Online Gene Expression Profiling (GEPIA Web Tool)

The database GEPIA (http://gepia.cancerpku.cn/index.html) (28) was used to analyze the RNA expression data related to this study, based on The Cancer Genome Atlas (TCGA) and Genotype-Tissue Expression (GTEx) projects.

### Cell Culture and Transfection

Human glioma cell lines U87-MG (cat. no. CL-0238, authentication by STR profiling) and U251 (cat. no. Cl-0237, authentication by STR profiling) were purchased from Procell Life Science (Wuhan, China). Normal human astrocytes SVG P12 (NHAs, cat. no. 338577, authentication by STR profiling) were obtained from BeNa Culture Collection (Kunshan, China). U87-MG cells were cultured in DMEM (HyClone, Logan, UT, USA) supplemented with 10% fetal bovine serum (FBS) (HyClone, Logan, UT, USA). U251 cells were maintained in DMEM/F12 medium (HyClone, Logan, UT, USA), containing 10% FBS. NHAs were cultured in respective astrocyte growth media, supplemented with rhEGF, insulin, ascorbic acid, GA-1000, L-glutamine (Thermo Fisher Scientific, Carlsbad, CA, USA) and 5% FBS. Cells were cultured at 37°C in a humid atmosphere supplied with 5% CO2. For gene silencing experiments, the most efficient short-hairpin RNA (shRNA) sequence targeting lncRNA-H19 (NCBI reference sequence: NR_002196) was 5’-CGTGACAAGCAGGACATGA-3’. A scramble fragment 5’-TTCTCCGAACGTGTCACGT-3’ was used as negative control that had no significant homology to any human gene sequences. They were cloned into pLKD-CMV-EGFP-2A-Puro-U6-shRNA (Obio Technology, Shanghai, China). Then, stem-loop oligonucleotides (TTCAAGAGA) were synthesized and cloned into lentivirus-based vector LV3, and resulting plasmids were named as LV3-sh-H19 and LV3-NC, respectively. Lentivirus packaging system including recombinant LV3-sh-H19 plasmid or LV3-NC together with two packaging plasmids (psPAX2 and pMD2.G) was co-transfected into cells using Lipofectamine 2000 (Thermo Fisher Scientific, Carlsbad, CA, USA). Then, lentiviral particles were harvested from the media 48 hours after transfection, from centrifuged supernatant (4000 g, 10 minutes, 4°C), and lentiviral particles were purified with 0.45 mm cellulose acetate filters. The titer of concentrated lentivirus was determined via dilution, adopting fluorescent microscopy. MiRNA-200a mimics and inhibitors were synthesized by OBiO Technology Co. Ltd (Shanghai, China). pcDNA3.1 vectors expressing CDK6 or ZEB1 were obtained from OBiO Technology Co. Ltd (Shanghai, China). Glioma cells at logarithmic growth phase were transfected with Lipofectamine 2000 (Thermo Fisher Scientific, Carlsbad, CA, USA). U87-MG and U251 cell lines were further selected using puromycin after lentiviral transduction. Transfection/transduction efficiencies were determined using RT-qPCR. Cells were harvested 48 hours after transfection and used for further experiments.

### RNA Extraction and Quantitative PCR

According to the manufacturers’ protocols, total RNA was extracted from the tissues or cells using TRIzol^®^ reagent (Thermo Fisher Scientific, Carlsbad, CA, USA), while miRNAs were isolated using miRNA easy Mini kit (Qiagen, Shenzhen, China). RNA concentration and quality were determined using the absorbance at 260/280 nm by ultra-micro spectrophotometer (NanoDrop2000c, Thermo Fisher Scientific, Carlsbad, CA, USA). RNA was subjected to first-strand cDNA synthesis using PrimeScript™ RT Master Mix/Perfect Real Time (Takara, Dalian, China). To evaluate the levels of miR−200, a TaqMan MicroRNA Assay kit (Applied Biosystems, Foster City, CA, USA) was used, and qPCR was performed using the Applied Biosystem 7500 Real−Time PCR System. U6 was used for normalization of miRNA levels. To determine the expression of H19, CDK6 and ZEB1, qPCR was performed using Maxima SYBR Green/ROX qPCR Master Mix (Thermo Fisher Scientific, Carlsbad, CA, USA), and GAPDH gene was used as an internal reference. Program used for thermal cycler was as follows: 95°C for 5 mins, followed by 45 cycles of 95°C for 15 sec, 60°C for 20 sec and 72°C for 10 sec. The 2^–∆∆Ct^ method was used for data analysis, and each experiment was performed in triplicate. The following primers were used: GAPDH forward, 5’−GCAAGAGCACAAGAGGAAGA−3’ and reverse, 5’−ACTGTGAGGAGGGGAGATTC−3’; U6 forward, 5-CGCTTCGGCAGCACATATACTA-3, and reverse, 5-CGCTTCACGAATTTGCGTGTCA-3; H19 forward, 5’-ATCGGTGCCTCAGCGTTCGG-3’, and reverse, 5’-CTGTCCTCGCCGTCACACCG-3’; miR-200a forward, 5’-GCCGAGTGGTGCGGAGAGG-3’, and reverse, 5’-CTCAACTGGTGTCGTGGA-3’, CDK6 forward, 5’-CGGGATCCACCATGGAGAAGGACGGCCTG-3’, and reverse, 5’-CGGATCCATTGCTCAGGCTGTATTCAGCTCCGA-3’, ZEB1 forward, 5’-GCCAATAAGCAAACGATTCTG-3’, and reverse, 5’-TTTGGCTGGATCACTTTCAAG-3’.

### Luciferase Assay

Targetscan (www.targetscan.org) and LncBase Predicted v.2 (http://www.microrna.gr/LncBase) were employed to predict the potential binding sites of miR-200a on the transcripts of *H19*, *CDK6* and *ZEB1*. We used H306 pMIR-REPORT Luciferase as empty vector, luciferase reporters were constructed through cloning of 3’UTR of wild-type as well as genes mutant-type [H19-3UTR (2000bp), ZEB1-3UTR (2333bp) and CDK6-3UTR (1494bp)]. Then, these sequences were subcloned into the MluI and HindIII sites of the empty vector (Obio Technology, Shanghai, China).Fragments containing complementary sequences of miR-200a were cloned into luciferase reporter vector (OBiO Technology, Shanghai, China). U87-MG cells were co-transfected with correspondent luciferase vectors and miRNA-200a mimics/negative control (miR-NC) using Lipofectamine 2000 (Thermo Fisher Scientific, Carlsbad, CA, USA). After 48-h, luciferase activity was determined using dual luciferase reporter assay system (Promega, Madison, WI, USA).

### Cell Counting Kit−8 Assay

Transfected cells were inoculated onto 96-well plates (2×10^4^ cells per well) and incubated at 37°C at indicated time points. Cell proliferation rate was analyzed using CCK-8 assay kit (Biosharp, Guangzhou, China). Briefly, 20μl CCK-8 reagent was added into each well, and the assay was performed in triplicate. Cells were further incubated at 37°C for additional 2-4 hours. Absorbance was measured at 450 nm.

### Transwell Assay

Transwell chamber assay was carried out to determine cell migration and invasion. In migration assay, 48 hours after transfection, cells were diluted with serum-free medium into 3×10^4^ cells/ml and then added into the upper chamber (BD Biosciences, Franklin Lakes, NJ, USA) with a 8−µm pore size. In invasion assay, 50 μl of 0.2 μg/μl Matrigel was homogeneously dispersed (Sigma−Aldrich, St. Louis, MO, USA) in the Transwell chamber. After Matrigel was solidified, cells were diluted using serum-free medium to 1.5x10^5^ cells/ml, and 200μl of cell suspension was inoculated into the upper chamber of Transwell. A total of 500μl medium containing 10% FBS was added to the lower chamber of Transwell. Cells were cultured at 37°C for 24h. Subsequently, non-migrative/invasive cells were removed by cotton swab. Remaining cells were fixed with 4% paraformaldehyde for 15min and stained with 0.1% crystal violet for 20 min. The number of cells passing through the membrane filter were counted under microscope (magnification, x100; Olympus Corp, Tokyo, Japan).

### Western Blotting

U87-MG and U251 cells were collected and resuspended in ice-cold RIPA buffer. Cells were then lysed on ice for half an hour and centrifuged at 12,000 rpm for 30 min. Protein concentration was measured using BCA Protein Assay kit (Beyotime, Shanghai, China). Protein samples were diluted with loading buffer (Beyotime, Shanghai, China) and then heated at 100°C for 10 min. Equal amount of protein samples (20μg) were separated using a 10% sodium dodecyl sulfonate-polyacrylamide gel by electrophoresis. Protein content was then transferred onto PVDF membranes (Millipore, Billerica, MA, USA) and blocked with 5% not-fat milk at room temperature for 2 h. Blocked membranes were then incubated with anti-β-actin (1:2000; BioVision, Milpitas, CA, USA), anti-CDK6 (1:2000; Atlas Antibodies AB, Bromma, Sweden) or anti-ZEB1 (1:1000; Atlas Antibodies AB, Bromma, Sweden) at 4°C overnight. The following day, membranes were rinsed using 1×TBST and incubated with horseradish peroxidase-conjugated goat anti-mouse IgG (1:5000, Cell Signaling Technology, Danvers, MA, USA) or anti-rabbit IgG (1:5000, Cell Signaling Technology, Danvers, MA, USA) at room temperature for 2 h. Protein bands were visualized using an enhanced chemiluminescence kit (ECL, Solarbo Life Sciences, Beijing, China) by exposure to X-ray films.

### *In Vivo* Xenograft

Female BALB/C nude mice (4-5-week-old, ~19g) were obtained from The Laboratory Animal Research Centre of Chongqing Medical University (Chongqing, China). The mice were housed in temperature-controlled environment (22 ± 2˚C) with ~60% relative humidity, under a 12-h dark/light cycle with libitum access to food/water for at least three days prior to the experiments. Mice were injected with U251 cells transfected with sh-NC/sh-H19. Generally, a total of 5x10^6^ cells were diluted using 250μl PBS and well suspended, then subcutaneously injected into the back of mice. Mice with developing tumors were closely monitored (four times/week). Subsequently, 49 days following injection, the mice were sacrificed, and tumor tissues were isolated and evaluated. Tumor volume was calculated as follows: V (mm^3^) = (length x width^2^)/2.

### Statistical Analysis

All the data were presented as means ± standard error of mean and analysed using SPSS 17.0 (SPSS Inc., Chicago, IL, USA). The significance of differences was analysed using one-way analysis of variance (ANOVA) or the Student’s t-test. A student-Newman-Keuls test was performed post-ANOVA. The relationship between RNA levels was evaluated using Spearman’s correlation analysis. P<0.05 was considered to indicate a statistically significant difference.

## Results

### Up-Regulation of lncRNA H19 Is Detected in Glioma Tissues and Cell Lines

**Figure 1:**
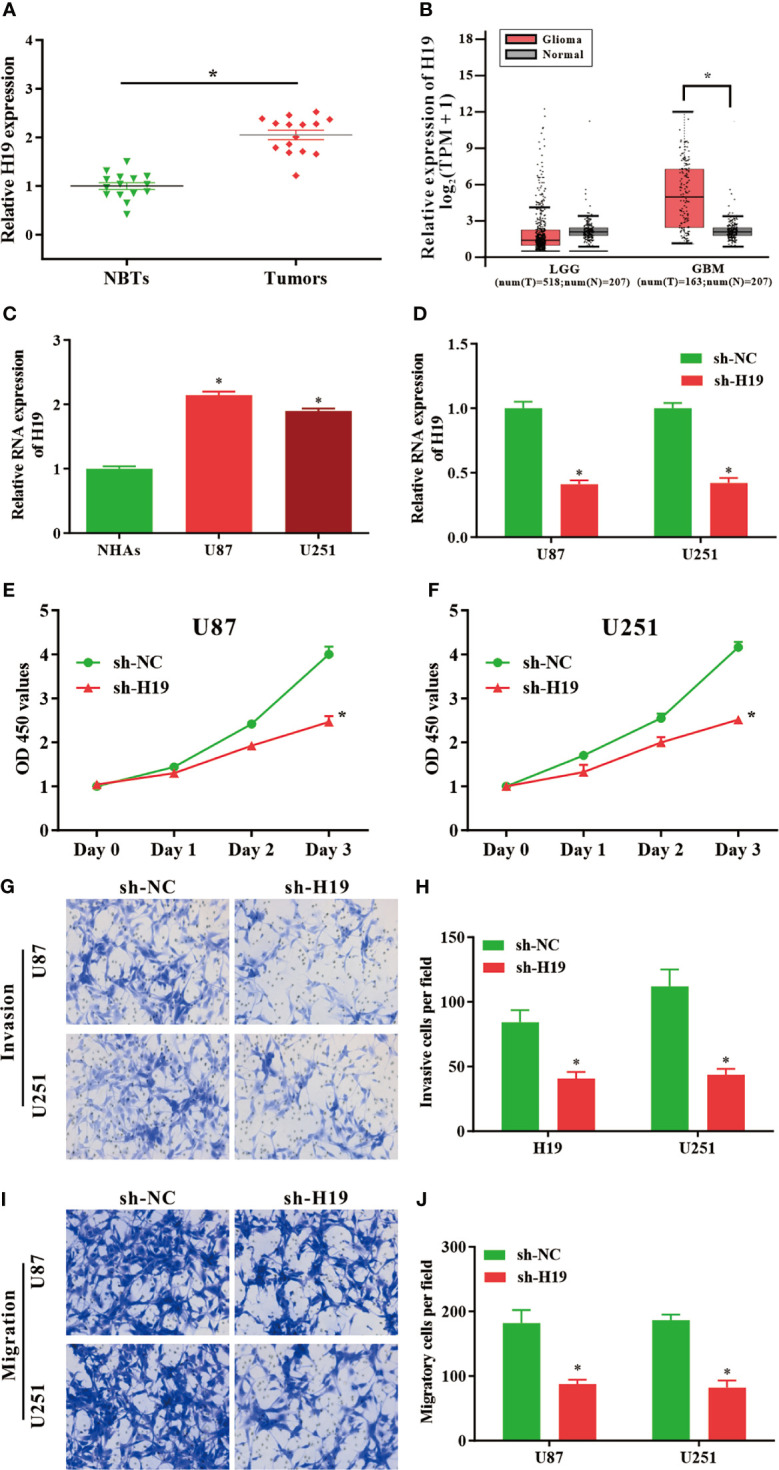
LncRNA H19 is significantly up-regulated in glioma tissues and cells, and down-regulation of H19 suppresses the proliferation, invasion and migration of glioma cells. **(A)** The RNA levels of H19 were determined by qRT-PCR. The expression of H19 were remarkably increased in glioma tissues compared with paired para−tumorous controls. **(B)** Analysis using GEPIA database revealed that H19 expression is significantly up-regulated in glioma tissues (GBM, T = 163, N = 207). Boxplot illustrated log2 (TPM + 1) on a log-scale; **(C)** The expression of H19 in NHAs and glioma cell lines (U87-MG and U251). **(D)** qPCR was performed to evaluate the transfection efficiency of sh-H19 in U87-MG and U251 cells. **(E, F)** The proliferative activities of U87-MG and U251 cells treated with sh-H19 or sh-NC were examined using CCK-8 assay. **(G, H)** The invasion of transfected cells were determined by Transwell assay (magnification, x100). **(I, J)** The migration of U87-MG and U251 cells treated with sh−H19 was also examined using Transwell assay (magnification, x100). Data were presented as mean ± SEM; *P < 0.05 *vs* NHAs or control group. NHAs, normal human astrocytes; NBTs, normal brain tissues; NC, negative control.

To investigate the expression of lncRNA H19 in glioma, the results of RT-qPCR confirmed that the RNA levels of H19 were notably upregulated in tumor samples compared with normal control (**Figure 1A**). And then GEPIA web tool was used to evaluate H19 levels in human specimens. The results revealed that H19 expression was remarkably up-regulated in glioma tissues (GBM) compared to noncancerous controls (Tumor=163, Normal=207, p<0.05). No significant difference was observed between low-grade gliomas (LGG) and para-carcinoma tissues (Tumor=518, Normal=207, p>0.05) (**Figure 1B**). Furthermore, qRT-PCR was performed to examine H19 expression in glioma cell lines (U87-MG and U251) compared to normal human astrocyte (NHAs). Our data indicated that H19 expression was elevated in U87-MG and U251 cells (p<0.001; **Figure 1C**). In summary, these results suggested that H19 was up-regulated in glioma tissues and cells.

### Knockdown of H19 Inhibits the Proliferation, Invasion, and Migration of Glioma Cells

To investigate the effects of H19 expression on the biological functions of glioma cells *in vitro*, U87-MG and U251 cells were transduced with shRNA-expressing lentiviruses targeting lncRNA H19 (sh-H19). The knockdown efficiencies were determined by RT-qPCR (**Figure 1D**). CCK8 assays revealed that the proliferative ability of U87-MG and U251 cells were decreased by the knockdown of H19 (**Figures 1E, F**). Data of transwell assay indicated that silenced H19 expression reduced the invasive (**Figures 1G, H**) and migratory (**Figures 1I, J**) capacities of glioma cells. These findings indicated that knockdown of endogenous H19 were able to suppress the malignant phenotype of glioma cells.

### H19 Acts as a Molecular Sponge of miR-200a and Regulates the Proliferation/Migration of Glioma Cells

**Figure 2:**
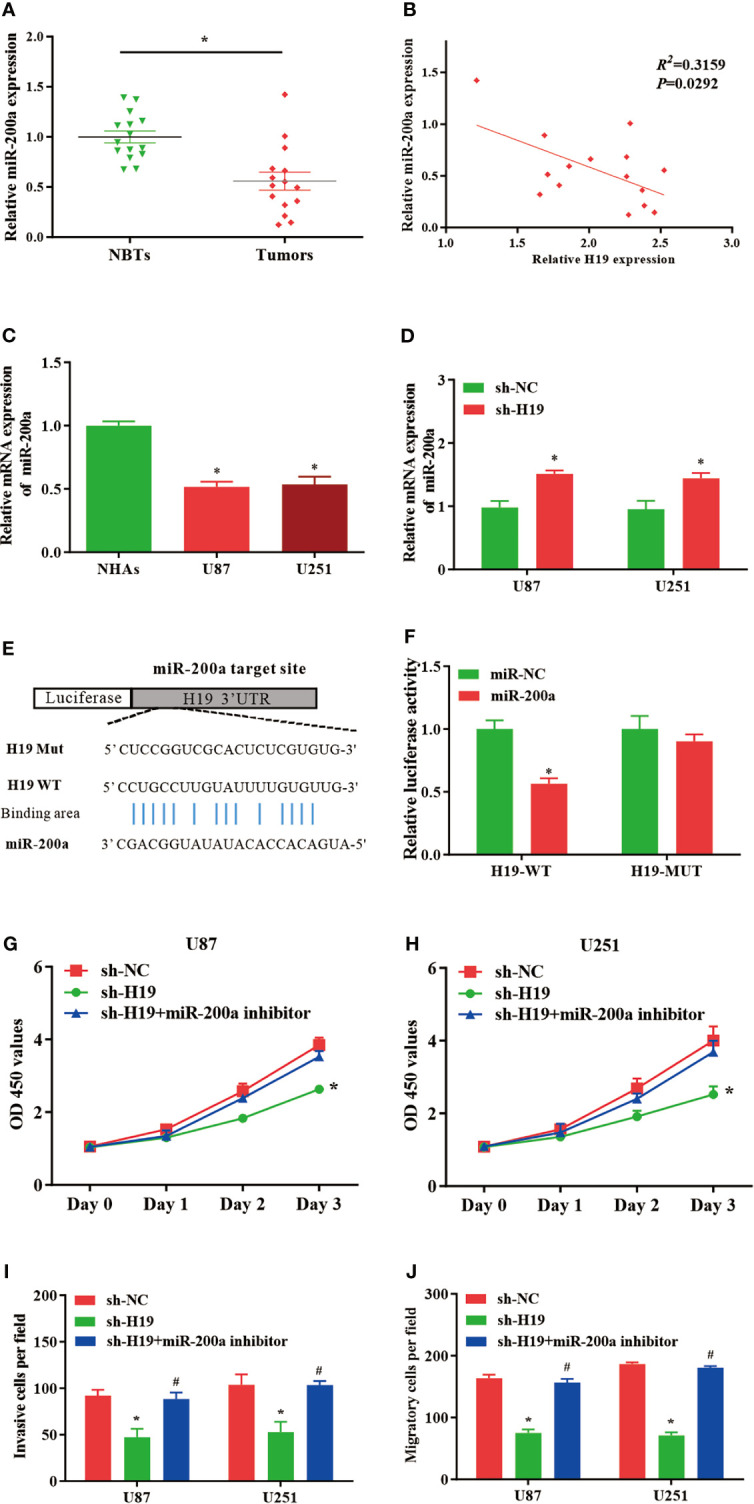
MiR-200a is a putative target gene of H19 in glioma cells. **(A)** The expression levels of miR−200a were examined in glioma tissues and paired para−tumorous controls using RT−qPCR. **(B)** The RNA levels of H19 and miR−200a inversely correlated in glioma tissues (R^2^ = 0.3159; P=0.0292). **(C)** The expression levels of miR-200a in normal human astrocytes (NHAs), U87-MG and U251 cells. **(D)** RT−qPCR was performed to evaluate the miR-200a expression in transfected U87-MG and U251 cells. **(E)** Potential binding sites between miR-200a and lncRNA H19 were predicted. **(F)** Overexpression of miR-200a decreased H19-WT-dependent luciferase activity, while H19-MUT-dependent luciferase signal remained unchanged. **(G, H)** The proliferation of transfected U87-MG and U251 cells was evaluated by CCK8 assay. **(I, J)** The invasion and migration of transfected cells were determined using Transwell assays. Data were presented as mean ± SEM; *P<0.05 *vs* NHAs or control group. ^#^ *P*<0.05 *vs* sh-H19 group. NHAs: normal human astrocytes. NBTs, normal brain tissues. NC, negative control.

In comparison with normal control, the expression of miR-200a was remarkably reduced in glioma samples, where the RNA levels of H19 and miR-200a were inversely correlated according to Spearman’s correlation analysis (**Figures 2A, B**). Furthermore, qPCR was performed to further evaluate the levels of miR-200a in glioma cells. The data indicated that miR-200a expression was decreased in glioma cells compared to normal human astrocytes (**Figure 2C**). The expression levels of miR-200a were elevated in U87-MG and U251 cells treated with sh-H19 (**Figure 2D**). To investigate whether H19 interacts with miRNAs, the putative binding sites of H19 on miR-200a were predicted using bioinformatic analysis (TargetScan, http://www.targetscan.org/) (**Figure 2E**). Wild-type (H19-WT) and mutant (H19-MUT) H19 sequences were further integrated into luciferase reporter plasmid. Luciferase reporter assays were then performed, and the results revealed that up-regulation of miR-200a lead to significant reduced luciferase activity in H19-WT transfected cells, but not in H19-MUT group (**Figure 2F**). Furthermore, U87-MG and U251 cells were transfected with sh-H19 alone or co-transfected with miR-200a inhibitors. Knockdown of H19 was able to suppress the proliferation, invasion and migration of glioma cells, which were abolished by the treatment with miR-200a inhibitors (**Figures 2G–J**). These data suggested that H19 could act as a molecular sponge of miR-200a, subsequently affecting the growth of glioma cells.

### CDK6, a Target Gene of miR−200a, Reverses the Biological Behavior Changes Caused by miR−200a Mimics

**Figure 3:**
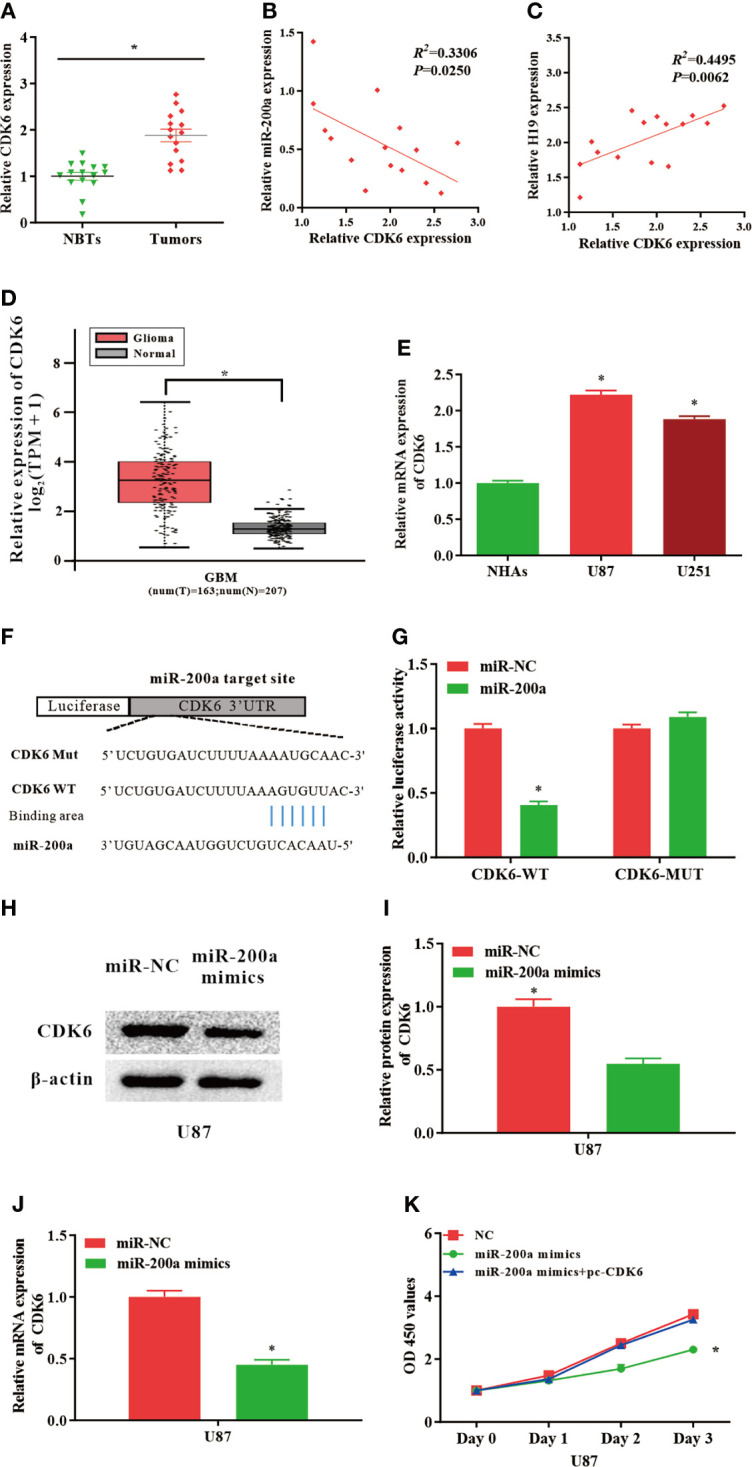
CDK6 levels are regulated by miR−200a in glioma cells. **(A)** CDK6 expression was assessed in glioma tissues and paired para−tumorous. **(B)** The RNA levels of CDK6 and miR−200a inversely correlated in glioma tissues (R^2^ = 0.3306; P = 0.0250). **(C)** The RNA levels of CDK6 and H19 correlated in glioma tissues (R^2^ = 0.4495; P = 0.0062). **(D)** GEPIA database revealed that CDK6 expression is remarkably up-regulated in glioma tissues (GBM, T = 163, N = 207). Boxplot illustrated log2 (TPM+1) on a log-scale. **(E)** CDK6 expression was up-regulated in glioma cells (U87-MG and U251) compared with NHAs. **(F)** Putative binding sites between miR-200a and CDK6 transcripts. **(G)** Overexpression of miR-200a lead to significant reduction of CDK6-WT-dependent luciferase activity, whereas no change was detected in mutant control. **(H, I)** Protein levels of CDK6 in U87-MG cells transfected with miR−NC/miR−200a mimics were determined by western blotting. **(J)** RT−qPCR was performed to evaluate the CDK6 expression in transfected U87-MG cells. **(K)** CCK-8 assay was performed to determine the proliferation of U87-MG cells treated with miR-200a mimics or co-transfected with miR-200a mimics and pc-CDK6. Data were presented as mean ± SEM; *P < 0.05 *vs* NHAs or control group. NHAs, normal human astrocytes; NBTs, normal brain tissues; NC, negative control.

Putative binding sequences between miR-200a and CDK6 were predicted by TargetScan database (**Figure 3F**). Furthermore, the interaction between miR-200a and CDK6 was confirmed using luciferase reporter assay. Our results revealed that miR-200a could significantly reduce wild-type CDK6-mediated luciferase activity, while the mutant group was not affected (**Figure 3G**). In comparison with normal control, the expression of CDK6 was notably upregulated in tumor samples compared with normal control (**Figure 3A**). The RNA levels of CDK6 and miR-200a were inversely correlated, while CDK6 and H19 were positively correlated according to Spearman’s correlation analysis (**Figures 3B, C**). In addition, by using GEPIA web tool, the up-regulation of CDK6 was further confirmed in glioma samples (GBM) compared to noncancerous tissues (Tumor=163, Normal=207, p<0.05; **Figure 3D**). In consistence with these findings, CDK6 expression was increased in glioma cell lines (**Figure 3E**). Furthermore, western blot analysis revealed that the protein levels of CDK6 were reduced in U87-MG cells treated with miR-200a mimics (**Figures 3H, I**). QPCR was performed to further evaluate the levels of CDK6 in U87-MG transfected with miR-200a mimics. The result indicated that the levels of CDK6 were notably reduced (**Figure 3J**). Overexpression of miR-200a was able to suppress the proliferation of U87-MG cells, and these effects were reversed by the up-regulation of CDK6 (**Figure 3K**). Taken all together, miR-200a could inhibit the proliferation of glioma cells through targeting CDK6.

### ZEB1, a Novel Downstream Molecule of miR−200a, Abrogates the Regulatory Effects of miR−200a in Glioma Cells

**Figure 4:**
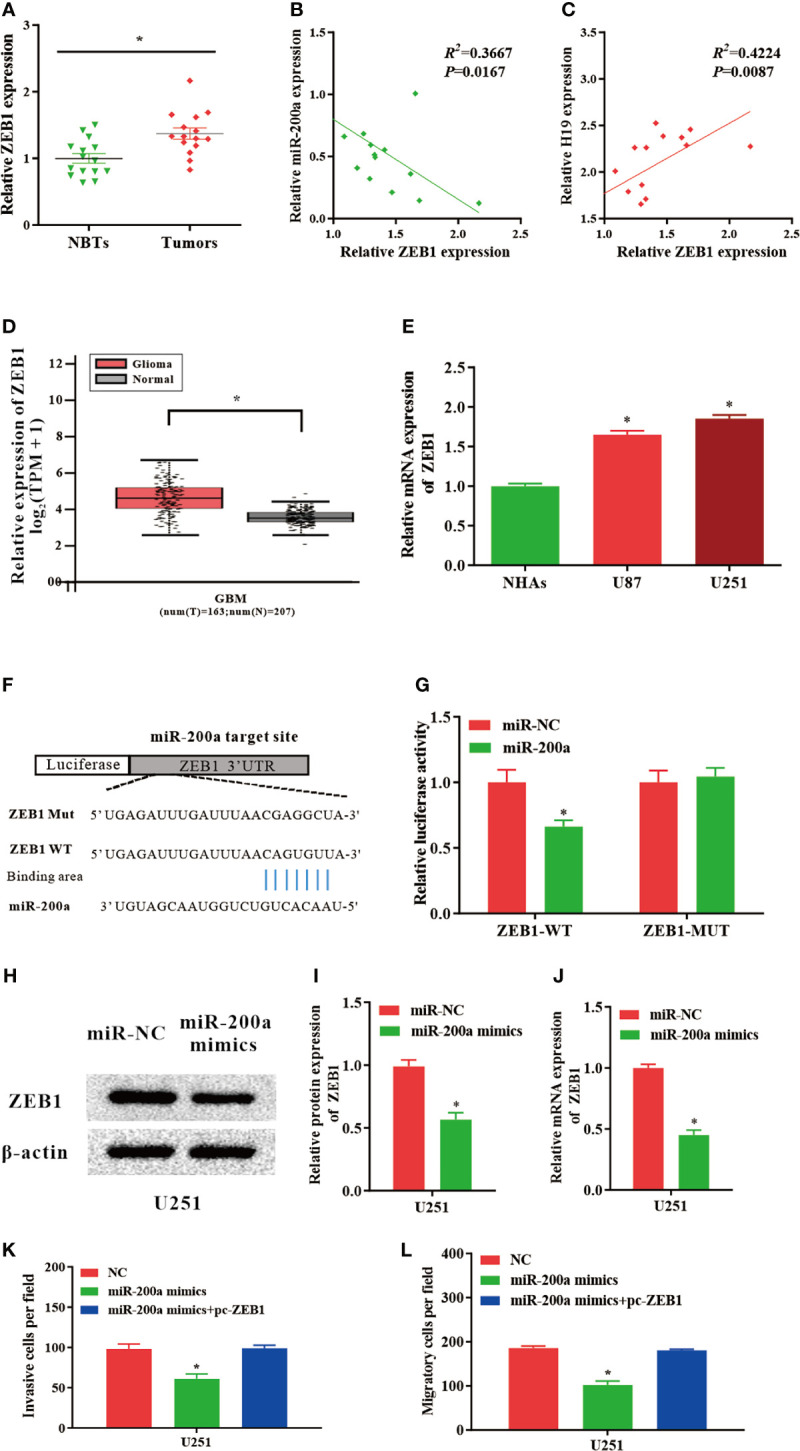
ZEB1 levels are modulated by miR−200a in glioma cells. **(A)** ZEB1 expression was assessed in glioma tissues and paired para−tumorous. **(B)** The RNA levels of ZEB1 and miR−200a inversely correlated in glioma tissues (R^2^ = 0.3667; P = 0.0167). **(C)** The RNA levels of ZEB1 and H19 correlated in glioma tissues (R^2^ = 0.4224; P = 0.0087). **(D)** GEPIA database revealed significant up-regulation of ZEB1 in glioma tissues (GBM, T = 163, N = 207). Boxplot illustrated log2 (TPM+1) on a log-scale. **(E)** ZEB1 expression was increased in glioma cell lines. **(F)** Putative binding sequences of miR-200a on ZEB1 transcript. **(G)** Overexpression of miR-200a resulted in significant decrease in ZEB1-WT-mediated luciferase activity but not in mutant control. **(H, I)** Protein levels of ZEB1 were determined in U251 cells transfected with miR-NC or miR-200a mimics. **(J)** RT−qPCR was performed to evaluate the ZEB1 expression in transfected U251 cells. **(K, L)** The invasion and migration of transfected U251 cells were examined using Transwell assay. Data were presented as mean ± SEM; *P < 0.05 *vs* NHAs or control group. NHAs, normal human astrocytes; NBTs, normal brain tissues; NC, negative control.

According to the databases, *ZEB1* was identified as a potential target gene of miR-200a (**Figure 4F**). Luciferase assays indicated that up-regulated miR-200a resulted in remarkably decreased luciferase activity in ZEB1-WT treated cells, but not in ZEB1-MUT group (**Figure 4G**). These findings suggested that ZEB1 is a novel target of miR-200a. Moreover, in comparison with normal control, the expression of ZEB1 was notably upregulated in tumor samples compared with normal control (**Figure 4A**). The RNA levels of ZEB1 and miR-200a were inversely correlated, while ZEB1 and H19 were positively correlated according to Spearman’s correlation analysis (**Figures 4B, C**). In addition, by using GEPIA web tool, the up-regulation of ZEB1 was further confirmed in glioma samples (GBM) compared to noncancerous tissues (Tumor=163, Normal=207, p<0.05; **Figure 4D**). In consistence with these findings, ZEB1 expression was increased in glioma cell lines (**Figure 4E**). Western blotting indicated that the protein levels of ZEB1 were reduced in U251 cells transfected with miR-200a mimics (**Figures 4H, I**). Furthermore, qPCR revealed that the levels of ZEB1 were significantly downregulated in U251 cells treated with miR-200a mimics (**Figure 4J**). To further investigate whether ZEB1 is involved in miR-200a-mediated regulatory function in glioma cells, U251 cells were transfection with miR-200a mimics alone or co-transfected with pc-ZEB1. The results suggested that up-regulation of miR-200a was able to inhibit the invasion and migration of glioma cells, which were abolished by overexpressed ZEB1 (**Figure 4K, L**). In summary, miR-200a could suppress the invasion/migration of glioma cells via directly binding to ZEB1.

### Knockdown of H19 Inhibits the Tumor Progression of Glioma *In Vivo*

**Figure 5:**
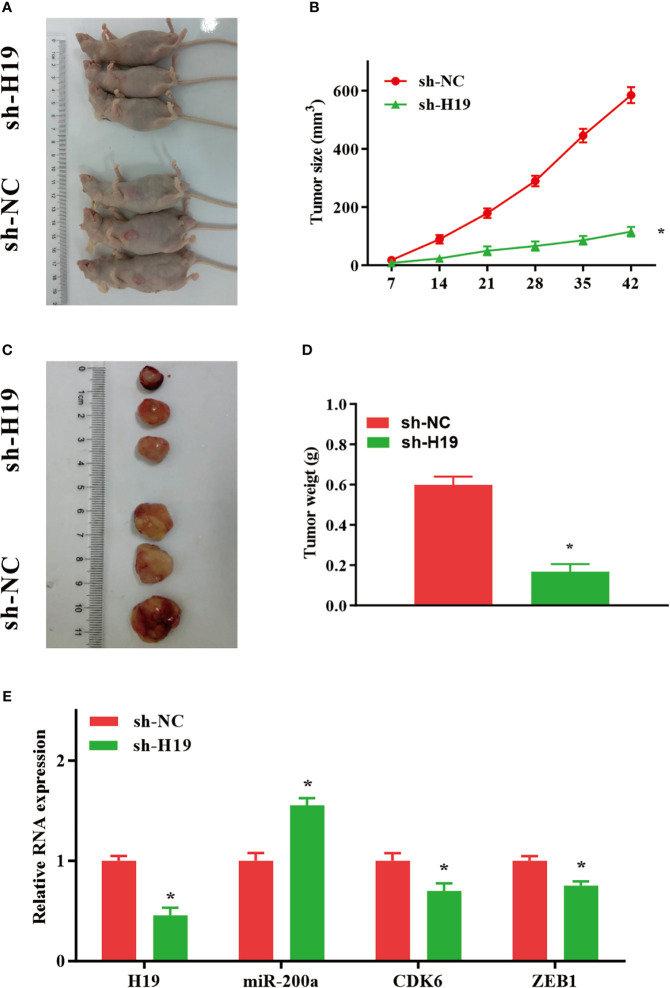
Knockdown of H19 suppressed the tumour progression of glioma in mouse model. **(A, C)** Seven weeks following injection, mice were sacrificed and tumor tissues were isolated. **(B, D)** The average tumour size and weight were remarkably decreased in H19 knockdown group compared with the control. **(E)** The levels of H19, CDK6 and ZEB1 were downregulated, and miR-200a was upregulated in H19 knockdown mice, respectively. Data were presented as mean ± SEM (*n* = 5 animals in each group); *P < 0.05 *vs* control group. NC, negative control.

To further elucidate whether H19 could affect the growth and metastasis of glioma *in vivo*, cells transfected with sh-NC or sh-H19 were injected into BALB/C nude mice subcutaneously. Seven weeks after injection, mice were sacrificed and tumor tissues were examined (**Figures 5A, C**). Furthermore, average tumor volume in sh-H19 mice was notably decreased compared with control group (**Figure 5B**). Additionally, mean values of tumor weight in H19 knockdown mice was remarkably reduced (**Figure 5D**). Moreover, RT-qPCR confirmed the downregulation of H19, CDK6 and ZEB1 in the knockdown mice, while the levels of miR-200a were significantly increased (**Figure 5E**). In summary, our data revealed that knockdown of H19 may suppress the development of glioma *in vivo* by up-regulating miR-200a and down-regulating CDK6/ZEB1.

## Discussion

LncRNAs are key regulators of chromosome remodeling and RNA metabolism (29). Numerous lncRNAs are associated with the occurrence and development of tumors, by acting as either oncogenic factors or tumor suppressors. The length of H19 gene was ~2.3 kilobases (kb), and it is located on chromosome 11p15. Luo et al. (30) have indicated that H19 expression is notably increased in bladder cancer specimens compared to para-carcinoma tissues. Tsang et al. (31) have suggested that the levels of H19 and miR-675 were significantly elevated in colorectal cancer. Yang et al. (32) have also revealed that H19 levels are increased in gastric cancer tissues, and knockdown of H19 lead to significant reduction on the invasiveness of gastric tumor. However, the detailed roles of H19 in tumor progression have not been fully understood, and its exact role in glioma has not been elucidated. Recently, there are several studies on the expression and functions of H19 in glioma. Zhou et al. (19) have suggested that H19 modulated the growth and metastasis of glioma cells by positively regulating the Wnt5a/β-catenin signaling pathway via directly targeting miR-342. Xiao et al. (18) have revealed that the expression of H19 was correlated with the degree of malignancy of glioma, indicating that H19 was a promising molecular marker for predicting the degree of glioma malignancy. Consistent with these previous studies, the results of our study revealed H19 expression was remarkably elevated in glioma. Furthermore, down-regulation of H19 could inhibit the malignant phenotype of glioma cells.

Previous studies have suggested that lncRNAs might function as “miRNA sponges” to suppress the expression of target miRNAs. In this study, H19 could regulate the growth and metastasis of glioma by targeting miR-200a. Moreover, miR-200a acts as a novel tumor suppressor gene and serves crucial roles on the onset and development of numerous types of tumors (33–35). For gliomas, Berthois et al (36) reported that the miR-200a expression level in grade IV glioma tissue was lower than those in grade II and III gliomas. Chen et al. (37) reported that miR-200a inhibits glioma cell survival, proliferation and invasion through the inhibition of FOXA1 expression. In consistence with these findings, our data revealed that H19 could enhance the aggressiveness of glioma cells by inhibiting the expression of miR-200a.

Furthermore, our result indicated that miR-200a may inhibit tumor progression by interacting with CDK6 and ZEB1. The data revealed that both CDK6 and ZEB1 were putative target genes of miR-200a. Accumulating evidence revealed the involvement of CDK6 in cancer pathogenesis, where it functions as a promising tumor promoter. Tang et al. (38) have evaluated tissue samples from 92 patients with pancreatic endocrine tumors, and the results indicated that CDK4/6 expression was increased in most of the tumor samples. Li et al. (39) have also revealed that CDK6 is significantly up-regulated in malignant glioma, which was closely associated with tumor progression. In the present study, the levels of CDK6 were remarkably elevated in glioma tissues and cells. Up-regulation of CDK6 was able to induce the proliferation of U87-MG cells, and vice versa, inhibition of CDK6 expression suppress the growth of glioma cells. Moreover, our data suggested that enhanced miR-200a expression lead to reduction of CDK6, subsequently inhibiting glioma cell proliferation. Furthermore, co-transfection pc-CDK6 could reverse the inhibitory effects caused by the miR-200a mimics. These results revealed that miR-200a could inhibit the malignant phenotype of glioma cells by down-regulating CDK6, and up-regulation of CDK6 could alleviate these inhibitory effects. Previous studies have also revealed that ZEB1 expression is increased in tumor tissues. For instance, ZEB1 expression was elevated in the tumor invasion front of gallbladder cancer tissues, which could be associated with the migration of tumor cells (40). Additionally, ZEB1 not only promotes the migration of tumor cells in pancreatic and colon cancer, but also serves essential roles on the initiation of tumor (41). Siebzehnrubl et al (42) revealed that *ZEB1* exerts simultaneous influence over invasion, chemoresistance and tumorigenesis in glioblastoma. Similarly, our results indicated that overexpressed miR-200a could suppress the migration and invasion of glioma cells by targeting ZEB1. Furthermore, miR-200a-mediated inhibitory effects were abolished by the overexpression of ZEB1. These data suggested that miR-200a might regulate the malignant phenotype of glioma cells *via* ZEB1.

**Figure 6:**
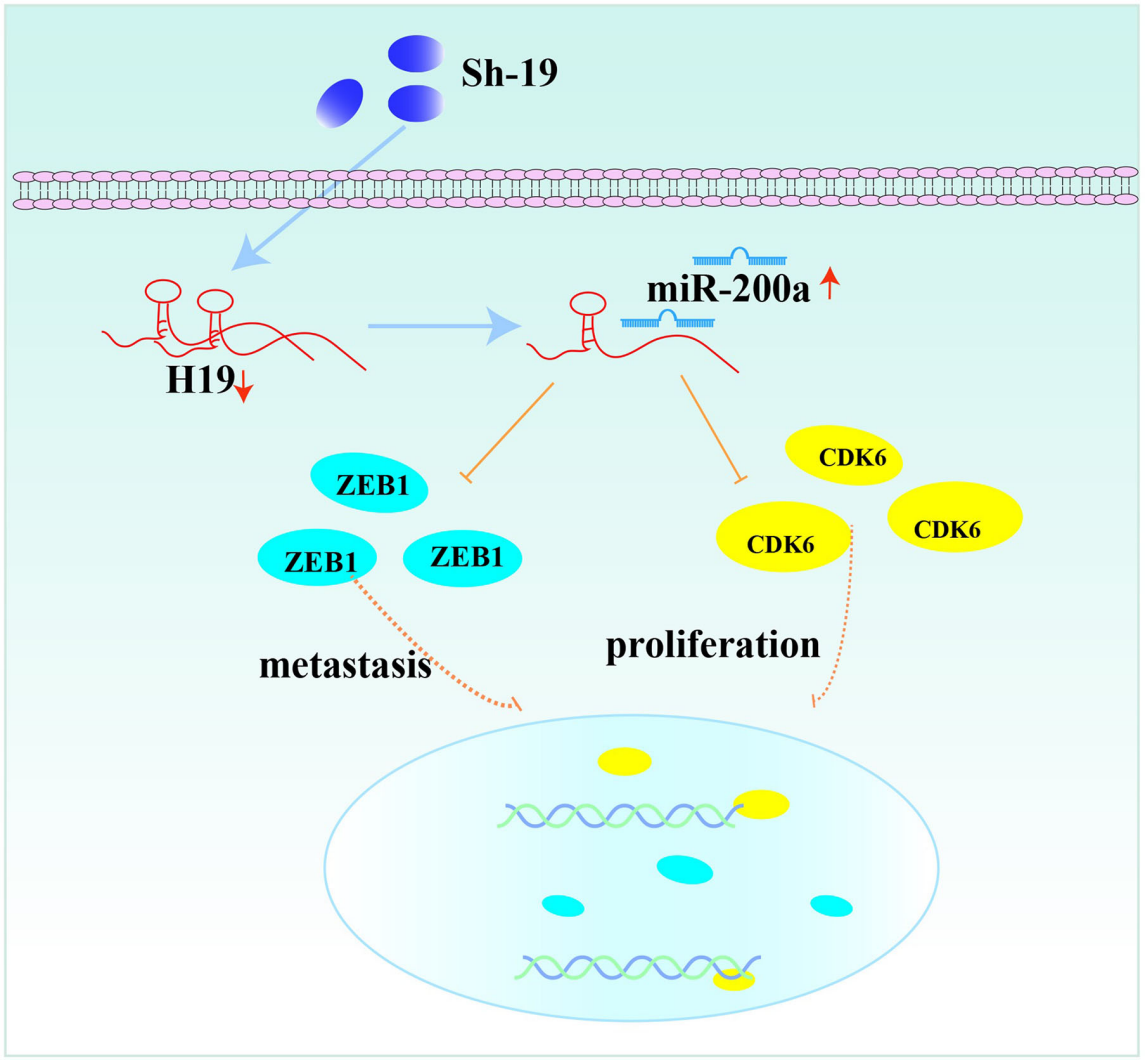
The feedback loop involving H19, miR-200a, CDK6 and ZEB1.

In conclusion, our results revealed the interaction of H19, miR-200a and CDK6/ZEB1 in glioma cells, which can affect tumor progression. Moreover, the novel feedback loop involving H19/miR-200a/CDK6 and ZEB1 could regulate the proliferation, invasion and migration of glioma cells (**Figure 6**). Knockdown of H19 could induce the biological behavior changes of glioma cells by up-regulating miR-200a and down-regulating CDK6/ZEB1. More importantly, these findings provide novel insights on future diagnosis and treatment of glioma.

## Data Availability Statement

The original contributions presented in the study are included in the article/**Supplementary Material**. Further inquiries can be directed to the corresponding authors.

## Ethics Statement

The animal study was reviewed and approved by Ethics Committee of the Third Military Medical University (Army Medical University).

## Author Contributions

All authors listed have made a substantial, direct, and intellectual contribution to the work.

## Funding

This work was supported by the National Natural Science Foundation of China (8217112662 and 4174D7). Funding bodies are not responsible for the study design, data collection/analysis, decision of publication or preparation of the manuscript.

## Conflict of Interest

The authors declare that the research was conducted in the absence of any commercial or financial relationships that could be construed as a potential conflict of interest.

## Publisher’s Note

All claims expressed in this article are solely those of the authors and do not necessarily represent those of their affiliated organizations, or those of the publisher, the editors and the reviewers. Any product that may be evaluated in this article, or claim that may be made by its manufacturer, is not guaranteed or endorsed by the publisher.

## Supplementary Material

Supplementary Figure 1
**(A–D)** The invasion and migration of transfected cells were determined using Transwell assays. Data were presented as mean ± SEM; **P* < 0.05 *vs* control group. ^#^ *P* < 0.05 *vs* sh-H19 group. NC, negative control.

Supplementary Figure 2
**(A–D)** The invasion and migration of transfected U251 cells were examined using Transwell assay. Data were presented as mean ± SEM; **P* < 0.05 *vs* control group. NC, negative control.
