# Supplementary figures and images for: Long Non−Coding RNA H19 Regulates Glioma Cell Growth and Metastasis via miR-200a-Mediated CDK6 and ZEB1 Expression
Source: Front Oncol. 2021 Nov 2;11:757650. doi: 10.3389/fonc.2021.757650 (PMC8593200; doi:10.3389/fonc.2021.757650)

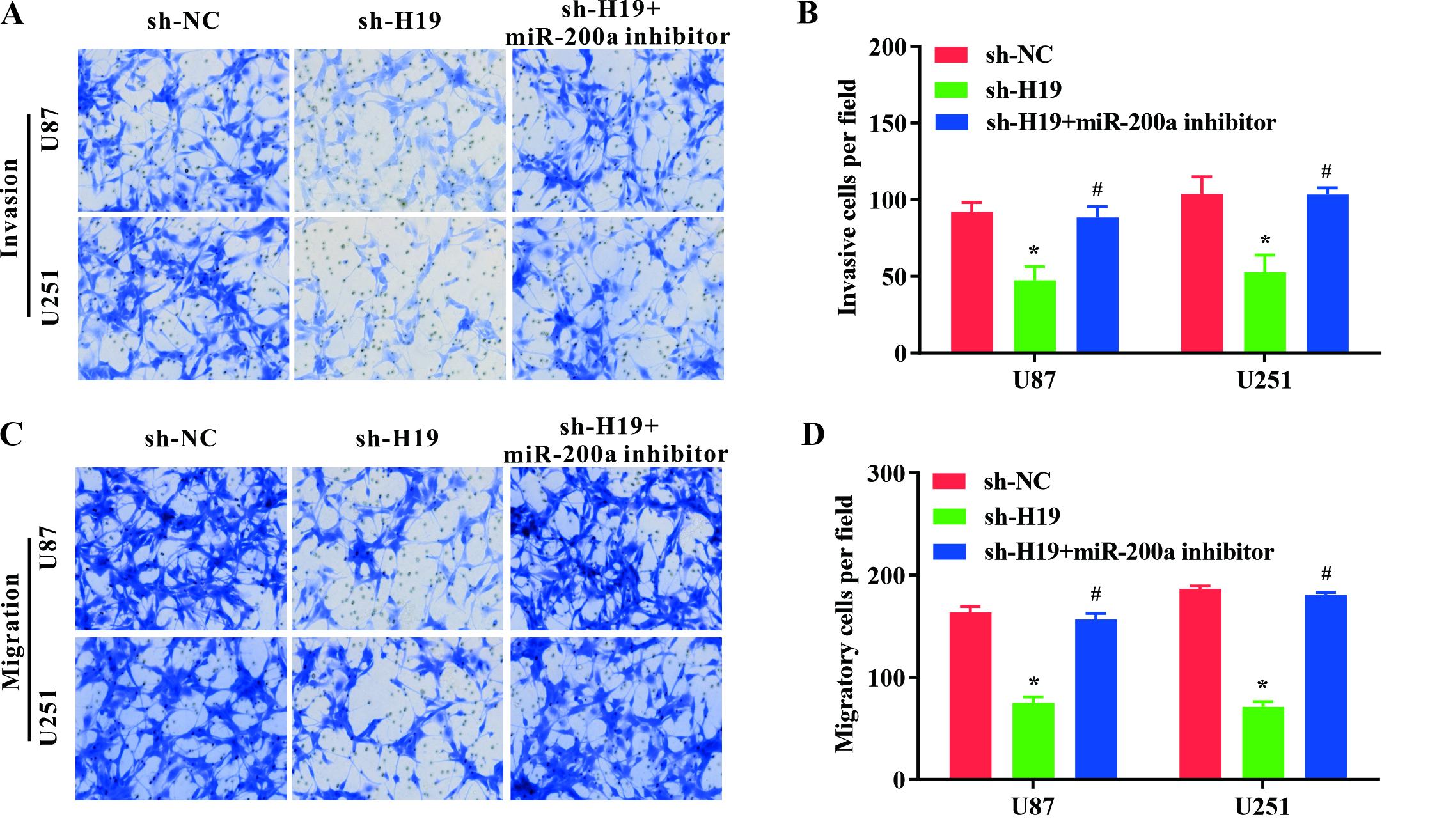

Supplement: Supplementary Figure 1 — (A–D) The invasion and migration of transfected cells were determined using Transwell assays. Data were presented as mean ± SEM; *P < 0.05 vs control group. # P < 0.05 vs sh-H19 group. NC, negative control. [file Image_1.tif]

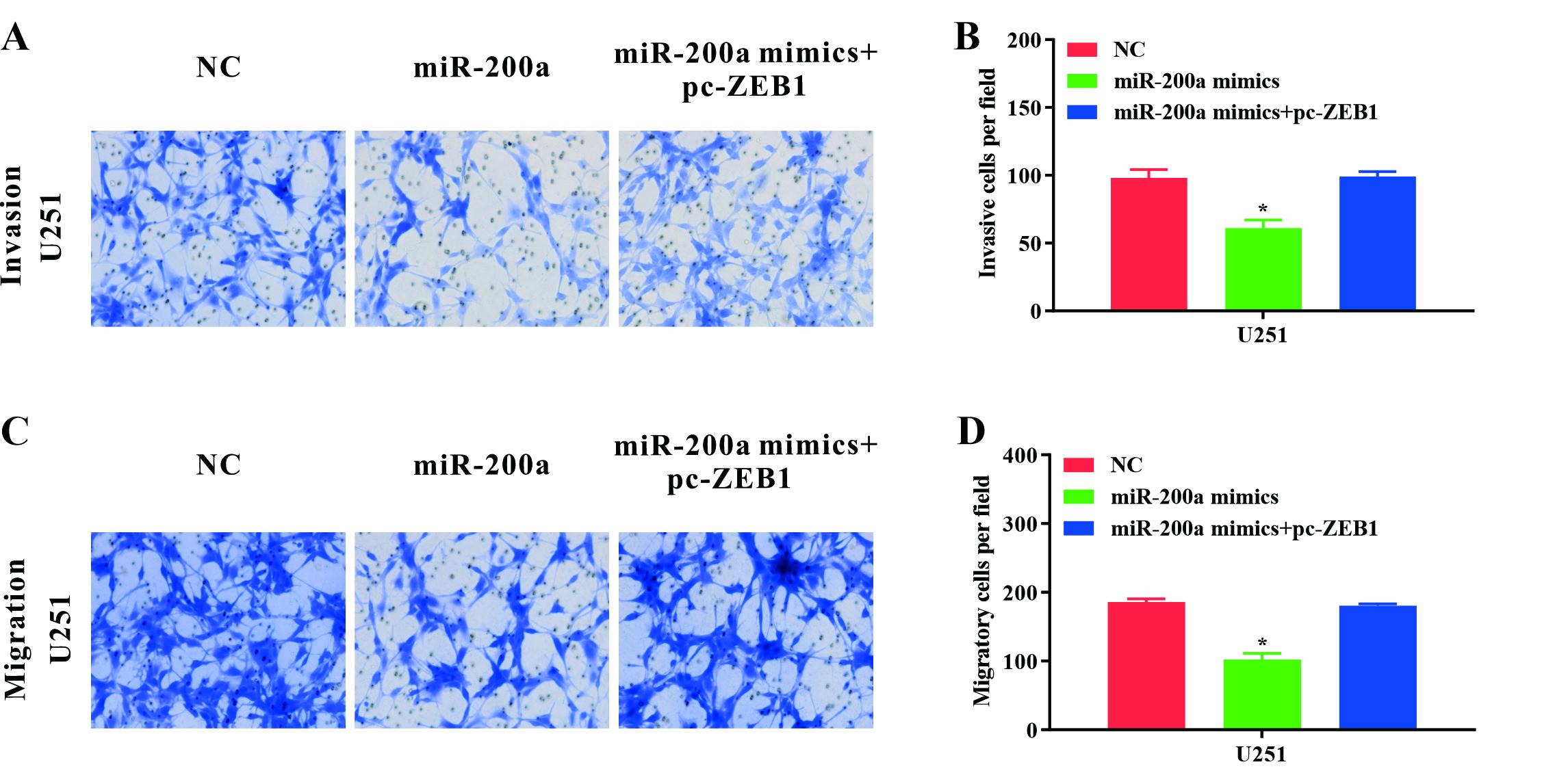

Supplement: Supplementary Figure 2 — (A–D) The invasion and migration of transfected U251 cells were examined using Transwell assay. Data were presented as mean ± SEM; *P < 0.05 vs control group. NC, negative control. [file Image_2.tif]
