# Supplementary material for: Long Non−Coding RNA H19 Regulates Glioma Cell Growth and Metastasis via miR-200a-Mediated CDK6 and ZEB1 Expression
Source: Front Oncol. 2021 Nov 2;11:757650. doi: 10.3389/fonc.2021.757650 (PMC8593200; doi:10.3389/fonc.2021.757650)
Supplement: Supplementary file 4 [file DataSheet_1.pdf]

Cell STR Certification Report

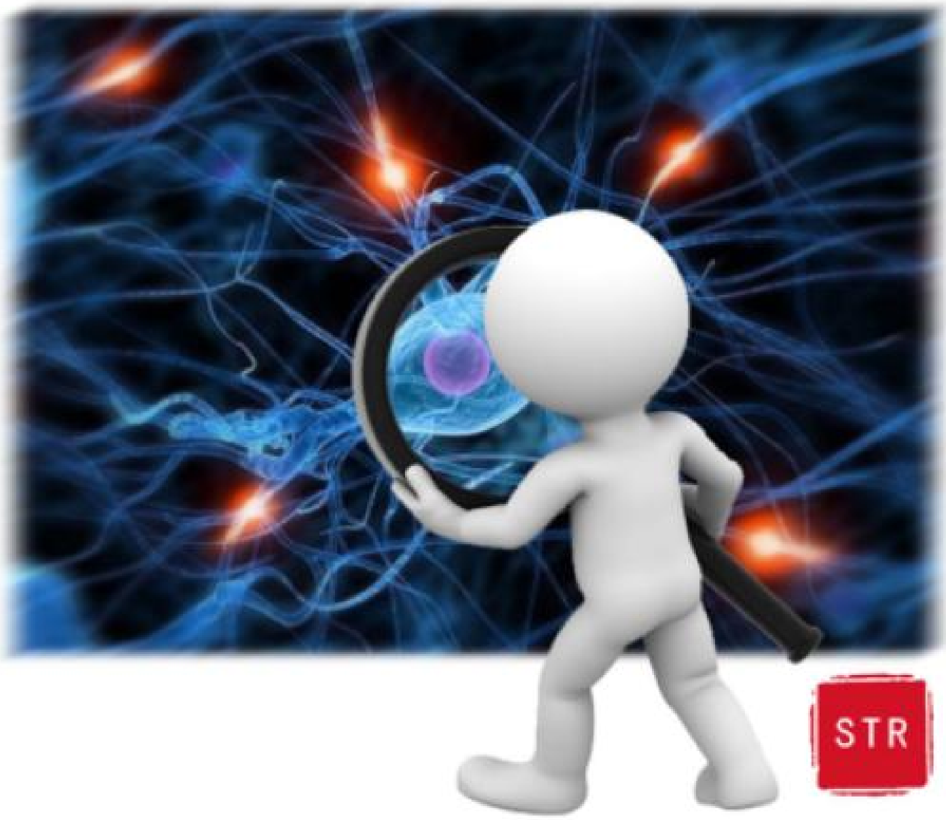

1、 Sample ID：SVG p12

2、 Original Material：Cell pellets

3、 Check time:2021-9-1

4、 Methods：

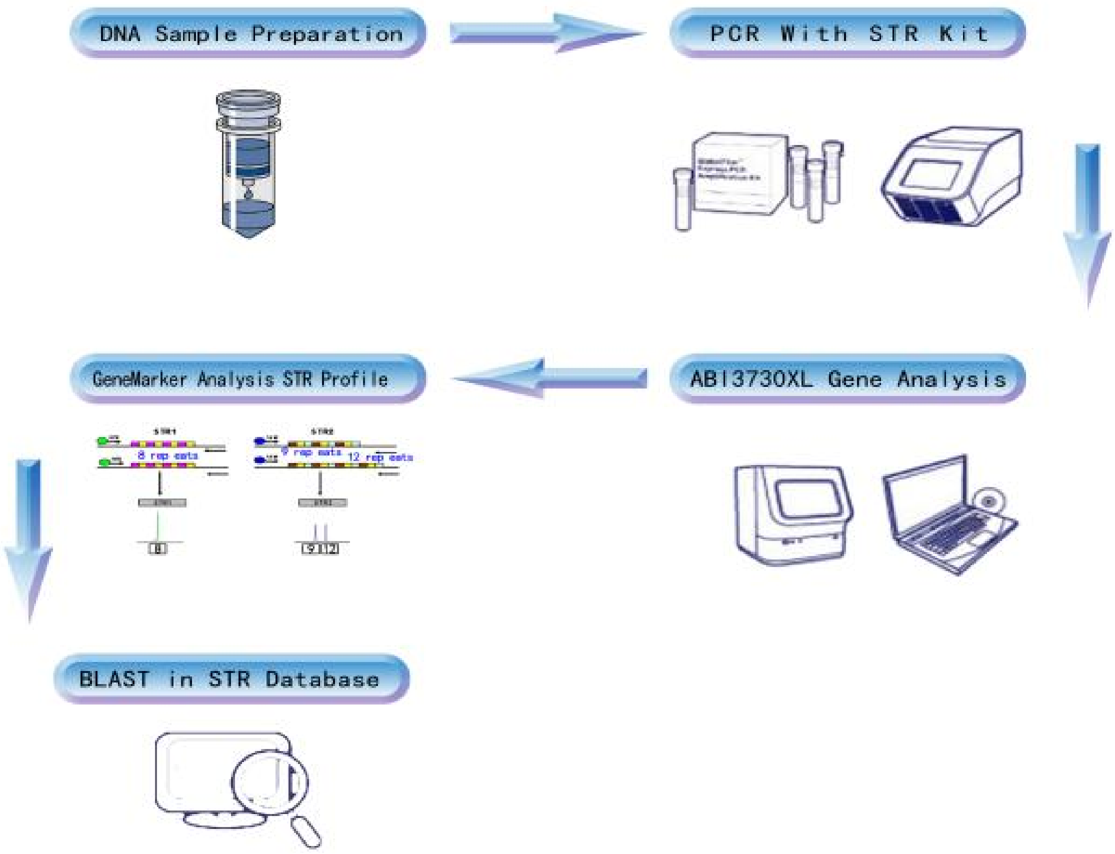

5、 Results：

Negative and positive test results are correct.

Amplification map of Genomic DNA clear，Genotyping results well.

STR Profile :

Genetic Site	Customer sample				**ATCC**			
	SVG p12				SVG p12			
Amelogenin	X		Y		X		Y	
CSF1PO	10		11		10		11	12
D13S317	8		13	14	8		13	14
D16S539	10		11	12	10		11	12
D5S818	11		12		11		12	14
D7S820	10		12	13	10		12	13
THO1	6		7	9.3	6		7	9.3
TPOX	8		10	11	8		10	11
vWA	15	16	18	19	15	16	18	19
Percent match between the sample and the database profile：96.1**%**								

6、 Summary：

The result of STR profile showed no more than 2 distinct alleles were found ,the sample derived from a common ancestry(Figure 1); which matched 96.1**%** the reference cell line in the **ATCC** STR database, named SVG p12.

Notes：

- P=100% x (2xM)/N; M: number of the matching peaks; N: number of all peaks
  For example:M=25, N=52, P=100% x (2x25)/52=**96.1%**
- Based on ASN-0002-2011 Standard， cell lines with ≥80% match are considered to be related ; i.e.,derived from a common ancestry. Cell lines with between a 55% to 80% match require futher profiling for authentication of relatedness.
- This data and analysis are for research use only.

Operator: Dehua Peng

Auditor: Jigang Tu

GUANGZHOU CELLBANK BIOTECH CO.,LTD
3 Sep. 2021
CELL STR PROFILING
PASS

Report time: 2021-9-3

Figure：

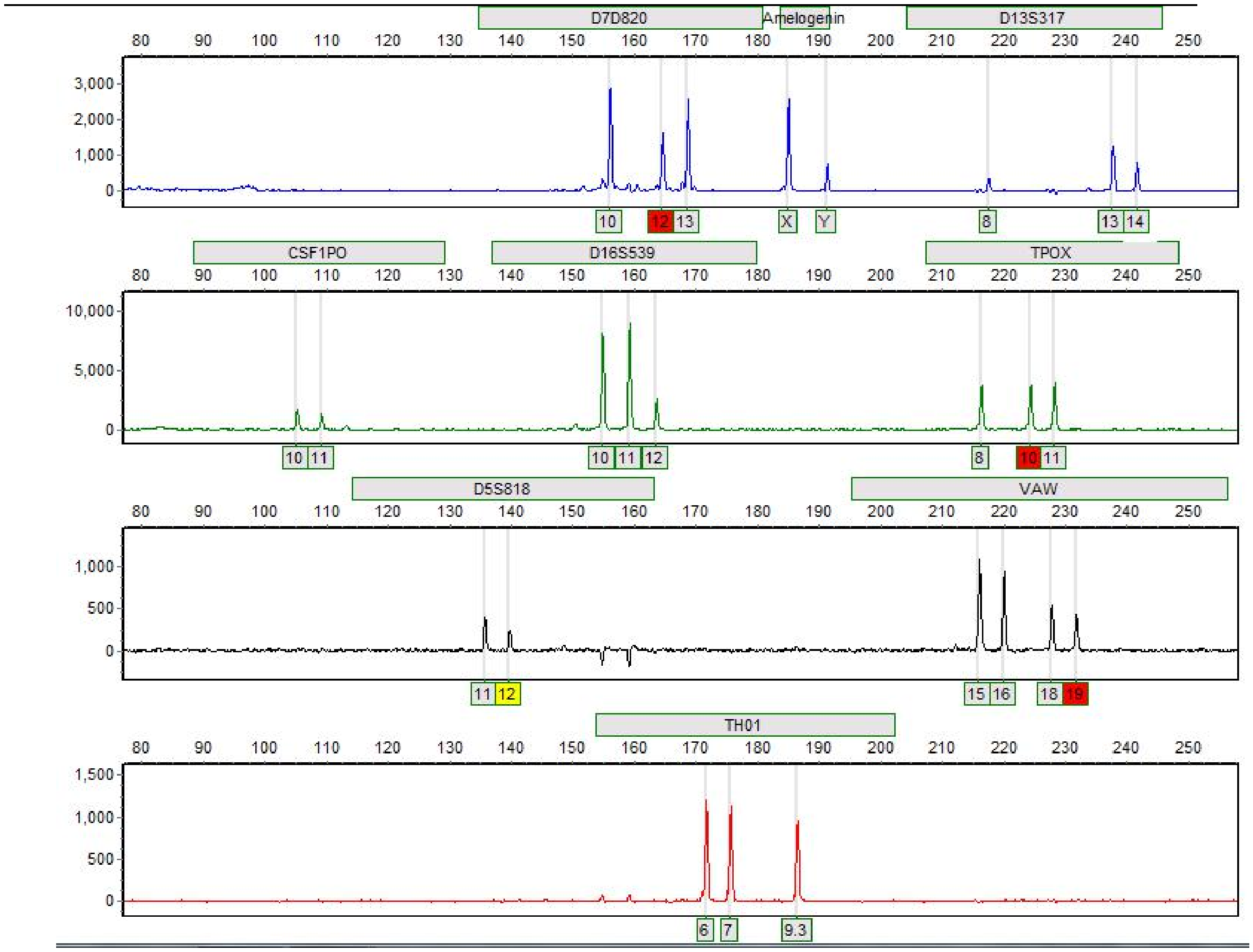

Figure 1.STR profiles of SVG p12 cell line
